# Supplementary material for: Intersectionality and benefit receipt: The interplay between education, gender, age and migration background
Source: PLoS One. 2024 Nov 14;19(11):e0311241. doi: 10.1371/journal.pone.0311241 (PMC11563431; doi:10.1371/journal.pone.0311241)
Supplement: S1 File — (PDF) [file pone.0311241.s001.pdf]

S1 Technical Description of the Analytical Strategy

In this appendix, we provide more detailed and technical information on the Multilevel Analyses of Individual Heterogeneity and Discriminatory Accuracy [MAIHDA: see 1] that were performed in this study. This appendix aims to provide additional information to facilitate the replication of similar models by explicating model configurations, prior distributions and formulas used of key statistics used in the paper.

We estimated Bayesian Multilevel Logistic Regression Models in `Stata 16.1`, using a Metropolis-Hastings algorithm for the Markov Chain Monte Carlo (MCMC) resampler. All models were estimated using 4 chains with a burn-in length of 10,000 iterations, after which we ran 10,000 iterations to estimate the posterior distributions. We used the posterior distribution of the first chain to calculate the average and 95% credibility interval for all model statistics. Information of the other chains were used for model diagnostics.

In the remainder of this appendix we explicate the prior distributions used formulas used for all model statistics such as (Log Likelihoods, ICCs, PCVs, and predicted incidences). In our models, we use narrow prior distributions, since these operate more reliably with logistic models and because they scale better to the odds ratio level. We informed our prior distributions using the data from our train-split.

S1.1 Baseline Model

For our baseline model, we estimated a random intercept model, where individuals (i) are nested in intersectional strata (j). In the realm of MAIHDA, these models are generally referred to as unadjusted models. In the realm of multilevel regression, these models are named variance components models. We configured our baseline model as:

$$
\begin{aligned}
\text{model:} \qquad && Y_{_{0ij}} &\sim \text{Bernouli}(\pi_{_{0ij}}) \\
&& \log\left(\tfrac{\pi_{0ij}}{1-\pi_{0ij}}\right) &= \beta_{0_{0ij}} + u_{0_{0j}} \\
\text{prior:} \qquad && \beta_{0_{0ij}} &\sim \text{cauchy}(-1, 4) \\
&& u_{0_{0j}} &\sim N(0, \sigma^2_{0j}) \\
&& \sigma^2_{0j} &\sim \text{igamma}(0.01, 0.01)
\end{aligned}
$$

Here Y_{ij} is the discrete outcome variable for SA or UI benefit receipt, which can only take values 0 or 1. We estimate the probability $\pi_{_{0ij}}$, which denotes the predicted probability of benefit receipt as a function of the overall intercept $\beta_{0_{0ij}}$ and the random intercepts per stratum $u_{0_{0j}}$. We calculate the predicted unadjusted incidences as:

$$\pi_{0ij} = \frac{e^{(\beta_{0_{0ij}} + u0_{0j})}}{1 + e^{(\beta_{0_{0ij}} + u0_{0j})}}$$

... and the ICC using a linearization approach proposed by Goldstein and colleague's [2] as:

$$\mathrm{ICC} = \frac{\sigma_{0j}^2 \cdot p^2(1+F)^{-2}}{p(1-p) + \sigma_{0j}^2 \cdot p^2(1+F)^{-2}}$$

... where F denote the odds ratios of the fixed effect predictions of the baseline model ($e^{\beta_{0_{0ij}}}$) and p denotes the predicted probability based on the fixed effects ($F/(1+F)$). This approach yields an advantage over conventionally used latent variable approaches for logistic models – where the first-level residual is fixed – since it is based on the mean distribution of the level 2 random effect.

S1.2 Partially Adjusted Models

For our partially adjusted models, we expanded our baseline model with including additive effect for each social dimension separately. The additive effects comprise dichotomous predictors for all but one social group per dimension. The left out social group serves as the reference category. We estimate models for gender, migration background, age, and education (respectively indexed: 1 – 4). Since we had an equivocal model specification for all partially adjusted models, we will illustrate our model configuration based on model 1 (in which we include additive effects of gender). The partially adjusted models were configured as:

$$\begin{aligned}
\text{model:} \quad & Y_{1ij} \sim \mathrm{Bernouli}(\pi_{1ij}) \\
& \log\left(\frac{\pi_{1ij}}{1-\pi_{1ij}}\right) = \beta_{0_{1ij}} + \beta_{1_{1ij}} X_{1ij} + u_{0_{1j}} \\
\text{prior:} \quad & \beta_{0_{1ij}} \sim \mathrm{cauchy}(-1, 4) \\
& \beta_{1_{1ij}} \sim \mathrm{cauchy}(0, 1.5) \\
& u_{0_{1j}} \sim N(0, \sigma_{1j}^2) \\
& \sigma_{1j}^2 \sim \mathrm{igamma}(0.01, 0.01)
\end{aligned}$$

In these models, a fixed effect of gender was included $\beta_{1_{1ij}}$ [1]. This changes the interpretation of the intercept parameter $\beta_{0_{1ij}}$ to the average incidence of people who belong to the reference category

[1] In the partially and fully adjusted models for SA, the prior distribution for migration background was configured as cauchy(0.5, 0.5), and cauchy(1.5, 0.5) and the constant prior for models that include migration background was configured as cauchy(-4, 0.5)

(in this case men). With the inclusion of the additive effects, the residual variance σ^2_{1j} might have shrunken compared to the baseline model. We calculated the Proportional Change of Variances using a bootstrapped approach as:

$$\text{PCV} = \frac{1}{B}\sum_{k=1}^{B}\left(\frac{\sigma^2_{0kj} - \sigma^2_{1kj}}{\sigma^2_{0kj}}\right)$$

... where B denotes the number of bootstrap samples of which we performed 10.000. Per bootstrap one random value k is sampled from the posterior distributions of σ^2_{0j} and σ^2_{1j}. Here the PCV represents how much of the variation at the stratum-level (i.e. ICC) can be explained by the additive effect of the social dimension (in this case gender).

S1.3 Fully Adjusted Models

For our fully adjusted models we included additive effects for all social dimensions that were used to construct the intersectional strata (indexed: 5). Per social dimension, dichotomous predictors were included for all social groups except the reference category. The fully adjusted model was configured as:

$$\begin{aligned}
\text{model:} \qquad & Y_{5ij} \sim \text{Bernouli}(\pi_{5ij}) \\
& \log\left(\frac{\pi_{5ij}}{1-\pi_{5ij}}\right) = \beta_{0_{5ij}} + \beta_{1_{5ij}} X_{5ij} + ... + \beta_{k_{5ij}} X_{5ij} + u_{0_{5j}} \\
\text{prior:} \qquad & \beta_{0_{5ij}} \sim \text{cauchy}(-1.5, 0.5) \\
& \beta_{1_{5ij}}, \beta_{k_{5ij}} \sim \text{cauchy}(0, 0.5) \\
& u_{0_{5j}} \sim N(0, \sigma^2_{5j}) \\
& \sigma^2_{5j} \sim \text{uniform}(0, 0.3)
\end{aligned}$$

Here $(\beta_{1_{5ij}}, \beta_{k_{5ij}})$ denote all the additive effect that were included in the model. Now, the random intercepts $u_{0_{5j}}$ capture the deviation of the additive incidence from the total predicted incidence and per intersectional stratum. To determine which stratum is relatively (dis-)advantaged, we (1) exponentiated the random intercepts to rescale them as odd ratios $\exp(u_{0_{5j}})$, next (2) we assert whether the 95% credibility intervals of exponentiated random intercepts did not include 1. Based on this model we calculated the total fully adjusted incidence rates as:

$$\pi_{5ij} = \frac{e^{(\beta_{0_{5ij}} + \beta_{1_{5ij}} + \, ... \, + \beta_{k_{5ij}} + u0_{5j})}}{1 + e^{(\beta_{0_{5ij}} + \beta_{1_{5ij}} + \, ... \, + \beta_{k_{5ij}} + u0_{5j})}}$$

... and the PCV using a bootstrapped approach as:

$$\mathrm{PCV} = \frac{1}{B}\sum_{k=1}^{B}\left(\frac{\sigma_{0kj}^2 - \sigma_{5kj}^2}{\sigma_{0kj}^2}\right)$$

References

1. Axelsson Fisk S, Mulinari S, Wemrell M, Leckie G, Perez V, Merlo J. Chronic obstructive pulmonary disease in Sweden: An intersectional multilevel analysis of individual heterogeneity and discriminatory accuracy. SSM - Population Health. 2018;4: 334–346. doi:10.1016/j.ssmph.2018.03.005

2. Goldstein H, Browne W, Rasbash J. Partitioning variation in multilevel models. Understanding Statistics. 2002;1: 223–231. doi:10.1207/S15328031US0104_02
