# Supplementary material for: Intersectionality and benefit receipt: The interplay between education, gender, age and migration background
Source: PLoS One. 2024 Nov 14;19(11):e0311241. doi: 10.1371/journal.pone.0311241 (PMC11563431; doi:10.1371/journal.pone.0311241)
Supplement: S2 File — (PDF) [file pone.0311241.s002.pdf]

S2 MAIHDA Results

S2.1 Variance Decompositioning

For social assistance, we find that 11.3% of the variance can be attributed to differences between intersectional groups. With our partially adjusted models (indexed 1 through 4), we included dummy variables per dimension to assess how much of the between strata variation in social assistance benefit receipt can be explained by each dimension. We find that differences between migration background groups (PCV = 0.359) and differences in education (PCV = 0.470) contributed considerably to the differences in social assistance receipt between intersectional groups. There are no substantial difference in social assistance receipt between men and women (PCV = -0.021) and age-groups (PCV = 0.008). In Model 5 we included the additive effects of all social dimensions, the PCV shows that almost all the between strata variance can be explained using an additive model (PCV = 0.793). This means that although a substantial amount of the differences in social assistance receipt can be attributed to intersectional group differences, just a small fraction (20.7%) of these differences are resulting from the complex combinations of social dimension related (dis-)advantages.

For unemployment insurance, we find that 4.1% of the incidence can be attributed to between strata differences. This shows that there is less variation in unemployment insurance incidence between intersectional groups compared to social assistance. This implies that other factors contribute more to whether someone received unemployment insurance. For all partially adjusted models (Model 1 – Model 4) the PCV was not different from 0, which means that for neither social dimension the additive effects

Table S1: ICC and PCV of Social Assistance and Unemployment Benefit Receipt

	Gender	Migration Background	Age	Education	Full
	(1)	(2)	(3)	(4)	(5)
**Social assistance**					
PCV	-0.021<br>(-0.406;0.279)	0.359<br>(0.115;0.546)	-0.008<br>(-0.382;0.280)	0.470<br>(0.269;0.627)	0.793<br>(0.743;0.838)
ICC*			0.113<br>(0.090;0.140)		
**Unemployment insurance**					
PCV	-0.010<br>(-0.402;0.299)	0.094<br>(-0.274;0.363)	0.239<br>(-0.053;0.475)	0.240<br>(-0.054;0.474)	0.517<br>(0.317;0.670)
ICC*			0.041<br>(0.032;0.052)		

* Based on the baseline model (Model 0)
Note: PCV: Averages of the Proportional Change of Variance and ICC: Intra-class Correlations posterior distributions, 95% credibility interval between parentheses. N(individuals) = 45,119. (Full, i.e. all additive effects). **Source:** Authors' own calculation based on non-public individual level register data from the Social Statistical Datasets (SSD) of Statistics Netherlands (CBS).

explained a substantial part of the between intersectional group differences in unemployment insurance receipt. In Model 5, the fully adjusted model, we find that 51.7% of the between stratum differences can be explained using solely additive effects. This means that even though there is limited variation between intersectional groups, a considerable fraction of this variation (48.3%) is caused by non-additive effects.

S2.2 Predicted Incidences

Table S2: Predicted Incidences per Intersectional Stratum for Social Assistance Benefit and Unemployment Insurance Benefit Receipt

Migration Background	Gender	Age	Education	SA	UI
Dutch	Female	Young	Academic	0.040	0.169
				(0.022;0.064)	(0.131;0.212)
			Non-Academic	0.056	0.213
				(0.033;0.083)	(0.172;0.258)
		Middle	Academic	0.014	0.208
				(0.004;0.028)	(0.165;0.253)
			Non-Academic	0.114	0.324
				(0.081;0.151)	(0.276;0.377)
		Old	Academic	0.025	0.254
				(0.011;0.045)	(0.210;0.303)
			Non-Academic	0.085	0.306
				(0.057;0.117)	(0.256;0.359)
	Male	Young	Academic	0.043	0.130
				(0.024;0.067)	(0.098;0.165)
			Non-Academic	0.062	0.192
				(0.039;0.092)	(0.152;0.236)
		Middle	Academic	0.019	0.168
				(0.008;0.036)	(0.131;0.210)
			Non-Academic	0.106	0.339
				(0.074;0.142)	(0.288;0.390)
		Old	Academic	0.028	0.256
				(0.013;0.048)	(0.207;0.305)
			Non-Academic	0.053	0.291
				(0.031;0.083)	(0.239;0.339)
Dutch Antillean 1st Gen.	Female	Young	Academic	0.062	0.193
				(0.038;0.093)	(0.152;0.238)
			Non-Academic	0.405	0.243
				(0.350;0.461)	(0.201;0.292)
		Middle	Academic	0.095	0.274
				(0.060;0.138)	(0.220;0.334)
			Non-Academic	0.496	0.381
				(0.438;0.556)	(0.328;0.435)
		Old	Academic	0.101	0.313
				(0.046;0.176)	(0.227;0.409)
			Non-Academic	0.430	0.347
				(0.375;0.485)	(0.293;0.400)
	Male	Young	Academic	0.044	0.167
				(0.022;0.075)	(0.126;0.215)
			Non-Academic	0.406	0.322
				(0.351;0.464)	(0.273;0.373)
		Middle	Academic	0.065	0.257
				(0.037;0.101)	(0.204;0.316)
			Non-Academic	0.431	0.386
				(0.375;0.485)	(0.336;0.438)
		Old	Academic	0.127	0.296
				(0.069;0.198)	(0.215;0.386)
			Non-Academic	0.401	0.399
				(0.345;0.454)	(0.344;0.454)
Dutch Antillean 2nd Gen.	Female	Young	Academic	0.049	0.173
				(0.029;0.075)	(0.137;0.215)
			Non-Academic	0.202	0.222
				(0.158;0.252)	(0.178;0.270)
		Middle	Academic	0.039	0.277
				(0.017;0.070)	(0.218;0.340)
			Non-Academic	0.170	0.353
				(0.130;0.215)	(0.303;0.405)
		Old	Academic	-	-

Table continues on next page ...

Continuation of Table					
**Migration Background**	**Gender**	**Age**	**Education**	**SA**	**UI**
			Non-Academic	0.136	0.321
				(0.072;0.222)	(0.233;0.422)
	Male	Young	Academic	0.043	0.162
				(0.023;0.067)	(0.125;0.202)
			Non-Academic	0.169	0.259
				(0.130;0.213)	(0.216;0.307)
		Middle	Academic	0.030	0.212
				(0.012;0.057)	(0.161;0.270)
			Non-Academic	0.173	0.359
				(0.132;0.216)	(0.305;0.411)
		Old	Academic	-	-
			Non-Academic	0.147	0.369
				(0.081;0.228)	(0.275;0.464)
EU 1st Gen.	Female	Young	Academic	0.034	0.192
				(0.018;0.056)	(0.151;0.238)
			Non-Academic	0.058	0.256
				(0.035;0.087)	(0.212;0.304)
		Middle	Academic	0.034	0.369
				(0.017;0.055)	(0.320;0.418)
			Non-Academic	0.137	0.415
				(0.100;0.179)	(0.362;0.473)
		Old	Academic	0.049	0.274
				(0.029;0.076)	(0.227;0.322)
			Non-Academic	0.181	0.314
				(0.139;0.226)	(0.264;0.366)
	Male	Young	Academic	0.038	0.226
				(0.019;0.062)	(0.184;0.273)
			Non-Academic	0.123	0.305
				(0.089;0.162)	(0.257;0.356)
		Middle	Academic	0.037	0.421
				(0.019;0.060)	(0.367;0.473)
			Non-Academic	0.126	0.461
				(0.091;0.166)	(0.406;0.518)
		Old	Academic	0.037	0.371
				(0.019;0.060)	(0.322;0.427)
			Non-Academic	0.159	0.443
				(0.122;0.202)	(0.388;0.496)
EU 2nd Gen.	Female	Young	Academic	0.050	0.177
				(0.028;0.076)	(0.139;0.219)
			Non-Academic	0.120	0.254
				(0.087;0.159)	(0.207;0.303)
		Middle	Academic	0.025	0.196
				(0.011;0.045)	(0.156;0.240)
			Non-Academic	0.179	0.370
				(0.135;0.224)	(0.314;0.422)
		Old	Academic	0.031	0.277
				(0.016;0.052)	(0.228;0.327)
			Non-Academic	0.111	0.346
				(0.077;0.147)	(0.295;0.399)
	Male	Young	Academic	0.034	0.150
				(0.017;0.058)	(0.112;0.191)
			Non-Academic	0.146	0.243
				(0.108;0.187)	(0.199;0.293)
		Middle	Academic	0.031	0.241
				(0.015;0.053)	(0.198;0.288)
			Non-Academic	0.114	0.333
				(0.079;0.151)	(0.283;0.385)
		Old	Academic	0.043	0.264
				(0.024;0.069)	(0.216;0.314)
			Non-Academic	0.078	0.388
				(0.050;0.109)	(0.334;0.441)
East EU 1st Gen.	Female	Young	Academic	0.062	0.299
				(0.037;0.092)	(0.247;0.351)
			Non-Academic	0.124	0.415

Table continues on next page ...

Continuation of Table					
**Migration Background**	**Gender**	**Age**	**Education**	**SA**	**UI**
				(0.090;0.160)	(0.363;0.466)
		Middle	Academic	0.077	0.380
				(0.050;0.108)	(0.328;0.429)
			Non-Academic	0.172	0.483
				(0.131;0.217)	(0.427;0.540)
		Old	Academic	0.158	0.300
				(0.114;0.210)	(0.245;0.360)
			Non-Academic	0.229	0.375
				(0.182;0.278)	(0.322;0.430)
	Male	Young	Academic	0.056	0.253
				(0.033;0.083)	(0.206;0.303)
			Non-Academic	0.062	0.419
				(0.038;0.089)	(0.364;0.472)
		Middle	Academic	0.044	0.451
				(0.024;0.071)	(0.391;0.512)
			Non-Academic	0.132	0.507
				(0.097;0.171)	(0.452;0.561)
		Old	Academic	0.141	0.407
				(0.081;0.214)	(0.319;0.498)
			Non-Academic	0.149	0.530
				(0.112;0.190)	(0.477;0.585)
East EU 2nd Gen.	Female	Young	Academic	0.051	0.181
				(0.028;0.081)	(0.138;0.230)
			Non-Academic	0.147	0.303
				(0.112;0.189)	(0.256;0.356)
		Middle	Academic	0.032	0.240
				(0.011;0.064)	(0.177;0.307)
			Non-Academic	0.160	0.361
				(0.120;0.200)	(0.310;0.413)
		Old	Academic	-	-
			Non-Academic	0.136	0.308
				(0.101;0.179)	(0.260;0.363)
	Male	Young	Academic	0.056	0.140
				(0.028;0.092)	(0.098;0.190)
			Non-Academic	0.126	0.235
				(0.091;0.167)	(0.192;0.283)
		Middle	Academic	0.031	0.190
				(0.011;0.062)	(0.137;0.253)
			Non-Academic	0.127	0.372
				(0.091;0.166)	(0.318;0.424)
		Old	Academic	0.061	0.284
				(0.019;0.124)	(0.192;0.388)
			Non-Academic	0.110	0.380
				(0.078;0.148)	(0.329;0.434)
Moroccan 1st Gen.	Female	Young	Academic	0.141	0.223
				(0.100;0.184)	(0.175;0.275)
			Non-Academic	0.362	0.278
				(0.309;0.417)	(0.228;0.330)
		Middle	Academic	0.305	0.378
				(0.250;0.363)	(0.323;0.436)
			Non-Academic	0.487	0.225
				(0.433;0.545)	(0.183;0.271)
		Old	Academic	-	-
			Non-Academic	0.596	0.121
				(0.541;0.650)	(0.090;0.158)
	Male	Young	Academic	0.106	0.302
				(0.070;0.148)	(0.246;0.362)
			Non-Academic	0.376	0.361
				(0.324;0.427)	(0.307;0.416)
		Middle	Academic	0.203	0.397
				(0.161;0.250)	(0.342;0.450)
			Non-Academic	0.403	0.375
				(0.349;0.459)	(0.323;0.427)

Table continues on next page ...

Continuation of Table					
**Migration Background**	**Gender**	**Age**	**Education**	**SA**	**UI**
		Old	Academic	0.299<br>(0.207;0.399)	0.362<br>(0.269;0.459)
			Non-Academic	0.430<br>(0.378;0.486)	0.370<br>(0.321;0.422)
Moroccan 2nd Gen.	Female	Young	Academic	0.059<br>(0.036;0.086)	0.210<br>(0.169;0.253)
			Non-Academic	0.239<br>(0.190;0.289)	0.281<br>(0.231;0.335)
		Middle	Academic	-	-
			Non-Academic	0.324<br>(0.272;0.380)	0.358<br>(0.306;0.414)
		Old	Academic	-	-
			Non-Academic	-	-
	Male	Young	Academic	0.101<br>(0.071;0.137)	0.199<br>(0.158;0.246)
			Non-Academic	0.350<br>(0.295;0.401)	0.265<br>(0.221;0.311)
		Middle	Academic	-	-
			Non-Academic	0.360<br>(0.309;0.413)	0.393<br>(0.341;0.445)
		Old	Academic	-	-
			Non-Academic	-	-
Other 1st Gen.	Female	Young	Academic	0.052<br>(0.031;0.080)	0.228<br>(0.183;0.276)
			Non-Academic	0.440<br>(0.382;0.497)	0.203<br>(0.161;0.247)
		Middle	Academic	0.153<br>(0.115;0.195)	0.315<br>(0.268;0.366)
			Non-Academic	0.402<br>(0.347;0.457)	0.296<br>(0.247;0.346)
		Old	Academic	0.192<br>(0.153;0.239)	0.271<br>(0.225;0.318)
			Non-Academic	0.471<br>(0.414;0.522)	0.204<br>(0.161;0.251)
	Male	Young	Academic	0.101<br>(0.070;0.139)	0.195<br>(0.155;0.242)
			Non-Academic	0.380<br>(0.325;0.435)	0.256<br>(0.211;0.305)
		Middle	Academic	0.263<br>(0.217;0.314)	0.331<br>(0.279;0.384)
			Non-Academic	0.428<br>(0.372;0.485)	0.355<br>(0.303;0.409)
		Old	Academic	0.297<br>(0.247;0.350)	0.317<br>(0.270;0.369)
			Non-Academic	0.414<br>(0.359;0.472)<br>(0.236;0.333)	0.359<br>(0.308;0.412)<br>(0.360;0.466)
Other 2nd Gen.	Female	Young	Academic	0.050<br>(0.029;0.077)	0.172<br>(0.132;0.214)
			Non-Academic	0.166<br>(0.125;0.207)	0.293<br>(0.245;0.342)
		Middle	Academic	0.016<br>(0.006;0.031)	0.238<br>(0.194;0.286)
			Non-Academic	0.126<br>(0.092;0.166)	0.361<br>(0.312;0.414)
		Old	Academic	0.025<br>(0.011;0.045)	0.244<br>(0.198;0.293)
			Non-Academic	0.101<br>(0.070;0.135)	0.324<br>(0.274;0.375)

Table continues on next page ...

Continuation of Table					
**Migration Background**	**Gender**	**Age**	**Education**	**SA**	**UI**
	Male	Young	Academic	0.040	0.163
				(0.022;0.064)	(0.124;0.204)
			Non-Academic	0.103	0.229
				(0.073;0.139)	(0.185;0.275)
		Middle	Academic	0.028	0.216
				(0.013;0.048)	(0.170;0.261)
			Non-Academic	0.128	0.384
				(0.092;0.164)	(0.328;0.439)
		Old	Academic	0.049	0.289
				(0.029;0.075)	(0.245;0.341)
			Non-Academic	0.114	0.435
				(0.082;0.151)	(0.382;0.492)
Surinamese 1st Gen.	Female	Young	Academic	0.071	0.202
				(0.046;0.101)	(0.159;0.247)
			Non-Academic	0.290	0.375
				(0.242;0.343)	(0.320;0.430)
		Middle	Academic	0.065	0.275
				(0.041;0.096)	(0.227;0.325)
			Non-Academic	0.356	0.416
				(0.304;0.410)	(0.362;0.471)
		Old	Academic	0.078	0.276
				(0.046;0.117)	(0.217;0.336)
			Non-Academic	0.290	0.336
				(0.241;0.342)	(0.286;0.391)
	Male	Young	Academic	0.047	0.177
				(0.024;0.080)	(0.132;0.229)
			Non-Academic	0.289	0.315
				(0.239;0.342)	(0.265;0.368)
		Middle	Academic	0.091	0.274
				(0.062;0.124)	(0.229;0.325)
			Non-Academic	0.311	0.419
				(0.261;0.366)	(0.365;0.474)
		Old	Academic	0.109	0.264
				(0.075;0.152)	(0.213;0.320)
			Non-Academic	0.284	0.410
				(0.236;0.333)	(0.360;0.466)
Surinamese 2nd Gen.	Female	Young	Academic	0.065	0.260
				(0.041;0.095)	(0.215;0.309)
			Non-Academic	0.280	0.357
				(0.232;0.330)	(0.305;0.412)
		Middle	Academic	0.046	0.217
				(0.027;0.072)	(0.177;0.263)
			Non-Academic	0.212	0.376
				(0.169;0.260)	(0.324;0.430)
		Old	Academic	-	-
			Non-Academic	0.199	0.387
				(0.151;0.249)	(0.325;0.446)
	Male	Young	Academic	0.056	0.189
				(0.033;0.086)	(0.149;0.233)
			Non-Academic	0.222	0.291
				(0.177;0.269)	(0.243;0.340)
		Middle	Academic	0.053	0.221
				(0.031;0.081)	(0.176;0.267)
			Non-Academic	0.294	0.412
				(0.244;0.347)	(0.358;0.464)
		Old	Academic	-	-
			Non-Academic	0.159	0.367
				(0.114;0.210)	(0.308;0.430)
Turkish 1st Gen.	Female	Young	Academic	0.062	0.269
				(0.038;0.091)	(0.224;0.317)
			Non-Academic	0.320	0.321
				(0.269;0.371)	(0.273;0.371)
		Middle	Academic	0.082	0.323

Table continues on next page ...

Continuation of Table					
**Migration Background**	**Gender**	**Age**	**Education**	**SA**	**UI**
				(0.053;0.117)	(0.271;0.378)
			Non-Academic	0.408<br>(0.357;0.459)	0.346<br>(0.294;0.402)
		Old	Academic	-	-
			Non-Academic	0.451<br>(0.398;0.512)	0.202<br>(0.160;0.244)
	Male	Young	Academic	0.059<br>(0.037;0.088)	0.261<br>(0.215;0.308)
			Non-Academic	0.190<br>(0.147;0.238)	0.389<br>(0.339;0.443)
		Middle	Academic	0.147<br>(0.109;0.189)	0.341<br>(0.292;0.391)
			Non-Academic	0.324<br>(0.272;0.377)	0.444<br>(0.390;0.496)
		Old	Academic	0.184<br>(0.116;0.263)	0.507<br>(0.414;0.596)
			Non-Academic	0.350<br>(0.298;0.404)	0.396<br>(0.343;0.452)
Turkish 2nd Gen.	Female	Young	Academic	0.087<br>(0.059;0.122)	0.219<br>(0.177;0.265)
			Non-Academic	0.225<br>(0.180;0.278)	0.326<br>(0.274;0.377)
		Middle	Academic	0.040<br>(0.012;0.089)	0.294<br>(0.209;0.389)
			Non-Academic	0.306<br>(0.254;0.358)	0.417<br>(0.362;0.471)
		Old	Academic	-	-
			Non-Academic	-	-
	Male	Young	Academic	0.065<br>(0.040;0.097)	0.150<br>(0.114;0.192)
			Non-Academic	0.231<br>(0.187;0.279)	0.319<br>(0.269;0.372)
		Middle	Academic	0.066<br>(0.027;0.124)	0.232<br>(0.162;0.319)
			Non-Academic	0.268<br>(0.219;0.317)	0.372<br>(0.321;0.426)
		Old	Academic	-	-
			Non-Academic	-	-

S2.3 Intersectional Effects

Table S3: 'Intersectional effects (AME) for Social Assistance Benefit Receipt and Unemployment Insurance Benefit Receipt'

**Migration Background**	**Gender**	**Age**	**Education**	**SA**	**UI**
Dutch	Female	Young	Academic	-0.138<br>(-0.195;-0.075)	-0.061<br>(-0.112;-0.006)
			Non-Academic	-0.080<br>(-0.089;-0.069)	-0.066<br>(-0.099;-0.030)
		Middle	Academic	-0.220<br>(-0.267;-0.165)	-0.101<br>(-0.155;-0.045)
			Non-Academic	-0.074<br>(-0.089;-0.057)	-0.064<br>(-0.104;-0.024)
		Old	Academic	-0.191<br>(-0.244;-0.131)	-0.011<br>(-0.068;0.045)
			Non-Academic	-0.081<br>(-0.095;-0.067)	-0.043<br>(-0.082;-0.001)
	Male	Young	Academic	-0.104<br>(-0.157;-0.043)	-0.129<br>(-0.174;-0.080)
			Non-Academic	-0.065<br>(-0.075;-0.054)	-0.099<br>(-0.129;-0.065)
		Middle	Academic	-0.178<br>(-0.226;-0.119)	-0.172<br>(-0.223;-0.120)
			Non-Academic	-0.061<br>(-0.076;-0.046)	-0.075<br>(-0.114;-0.034)
		Old	Academic	-0.156<br>(-0.209;-0.096)	-0.031<br>(-0.087;0.023)
			Non-Academic	-0.076<br>(-0.089;-0.063)	-0.075<br>(-0.111;-0.037)
Dutch Antillean 1st Gen.	Female	Young	Academic	-0.018<br>(-0.083;0.050)	0.013<br>(-0.039;0.072)
			Non-Academic	0.090<br>(0.060;0.120)	-0.009<br>(-0.046;0.031)
		Middle	Academic	0.032<br>(-0.050;0.118)	0.026<br>(-0.043;0.094)
			Non-Academic	0.133<br>(0.098;0.174)	0.026<br>(-0.017;0.071)
		Old	Academic	0.030<br>(-0.076;0.156)	0.077<br>(-0.018;0.171)
			Non-Academic	0.095<br>(0.062;0.131)	0.031<br>(-0.016;0.080)
	Male	Young	Academic	-0.040<br>(-0.102;0.034)	-0.038<br>(-0.100;0.025)
			Non-Academic	0.098<br>(0.068;0.130)	0.029<br>(-0.014;0.078)
		Middle	Academic	-0.008<br>(-0.085;0.073)	-0.013<br>(-0.080;0.057)
			Non-Academic	0.106<br>(0.073;0.141)	0.012<br>(-0.034;0.063)
		Old	Academic	0.088<br>(-0.021;0.207)	0.048<br>(-0.041;0.137)
			Non-Academic	0.091<br>(0.061;0.123)	0.054<br>(0.007;0.101)
Dutch Antillean 2nd Gen.	Female	Young	Academic	0.021<br>(-0.031;0.084)	0.012<br>(-0.042;0.071)
			Non-Academic	0.028<br>(0.011;0.046)	-0.007<br>(-0.041;0.028)
		Middle	Academic	-0.014<br>(-0.069;0.057)	0.051<br>(-0.016;0.123)
			Non-Academic	0.012<br>(-0.004;0.030)	0.027<br>(-0.021;0.072)
		Old	Academic	-	-

Table continues on next page ...

Continuation of Table					
**Migration Background**	**Gender**	**Age**	**Education**	**SA**	**UI**
			Non-Academic	0.002 (-0.018;0.029)	0.025 (-0.035;0.095)
	Male	Young	Academic	0.018 (-0.031;0.077)	-0.019 (-0.070;0.035)
			Non-Academic	0.021 (0.006;0.037)	0.004 (-0.030;0.041)
		Middle	Academic	-0.021 (-0.069;0.044)	-0.037 (-0.103;0.031)
			Non-Academic	0.019 (0.002;0.036)	0.015 (-0.029;0.060)
		Old	Academic	-	-
			Non-Academic	0.009 (-0.012;0.037)	0.045 (-0.021;0.123)
EU 1st Gen.	Female	Young	Academic	-0.053 (-0.100;0.007)	-0.042 (-0.098;0.014)
			Non-Academic	-0.034 (-0.043;-0.024)	-0.046 (-0.080;-0.007)
		Middle	Academic	-0.066 (-0.117;-0.008)	0.063 (0.005;0.122)
			Non-Academic	-0.016 (-0.031;-0.000)	-0.003 (-0.055;0.052)
		Old	Academic	-0.029 (-0.090;0.040)	0.003 (-0.055;0.061)
			Non-Academic	-0.000 (-0.018;0.018)	-0.046 (-0.088;-0.003)
	Male	Young	Academic	-0.029 (-0.078;0.032)	-0.025 (-0.080;0.031)
			Non-Academic	-0.008 (-0.020;0.006)	-0.029 (-0.070;0.012)
		Middle	Academic	-0.039 (-0.092;0.021)	0.087 (0.024;0.146)
			Non-Academic	-0.012 (-0.026;0.003)	0.012 (-0.039;0.067)
		Old	Academic	-0.040 (-0.090;0.022)	0.078 (0.019;0.139)
			Non-Academic	-0.000 (-0.016;0.018)	0.034 (-0.016;0.086)
EU 2nd Gen.	Female	Young	Academic	0.029 (-0.031;0.093)	-0.001 (-0.053;0.053)
			Non-Academic	0.004 (-0.012;0.018)	0.000 (-0.033;0.037)
		Middle	Academic	-0.035 (-0.083;0.018)	-0.058 (-0.114;-0.003)
			Non-Academic	0.019 (0.001;0.039)	0.021 (-0.024;0.066)
		Old	Academic	-0.021 (-0.073;0.033)	0.074 (0.013;0.133)
			Non-Academic	-0.003 (-0.019;0.012)	0.032 (-0.011;0.076)
	Male	Young	Academic	0.004 (-0.042;0.056)	-0.052 (-0.098;0.001)
			Non-Academic	0.016 (0.002;0.031)	-0.020 (-0.056;0.017)
		Middle	Academic	-0.009 (-0.056;0.046)	-0.027 (-0.084;0.034)
			Non-Academic	0.003 (-0.010;0.016)	-0.024 (-0.067;0.020)
		Old	Academic	0.020 (-0.034;0.078)	0.038 (-0.020;0.097)
			Non-Academic	-0.007 (-0.019;0.005)	0.047 (0.001;0.096)
East EU 1st Gen.	Female	Young	Academic	0.006 (-0.059;0.080)	0.044 (-0.010;0.105)
			Non-Academic	-0.017	0.041

Table continues on next page ...

Continuation of Table					
**Migration Background**	**Gender**	**Age**	**Education**	**SA**	**UI**
				(-0.031;-0.002)	(-0.005;0.092)
		Middle	Academic	0.027	0.032
				(-0.039;0.101)	(-0.026;0.089)
			Non-Academic	-0.007	0.018
				(-0.025;0.012)	(-0.037;0.075)
		Old	Academic	0.177	-0.008
				(0.087;0.268)	(-0.077;0.060)
			Non-Academic	0.014	-0.035
				(-0.005;0.034)	(-0.081;0.013)
	Male	Young	Academic	0.009	-0.031
				(-0.052;0.075)	(-0.086;0.029)
			Non-Academic	-0.028	0.024
				(-0.039;-0.017)	(-0.021;0.072)
		Middle	Academic	-0.028	0.075
				(-0.088;0.041)	(0.013;0.134)
			Non-Academic	-0.012	0.016
				(-0.026;0.004)	(-0.039;0.074)
		Old	Academic	0.129	0.072
				(0.019;0.251)	(-0.016;0.159)
			Non-Academic	-0.006	0.074
				(-0.021;0.011)	(0.020;0.129)
East EU 2nd Gen.	Female	Young	Academic	-0.005	0.027
				(-0.067;0.060)	(-0.024;0.083)
			Non-Academic	-0.004	0.055
				(-0.023;0.012)	(0.019;0.092)
		Middle	Academic	-0.055	0.019
				(-0.121;0.027)	(-0.055;0.100)
			Non-Academic	-0.005	0.045
				(-0.026;0.015)	(0.005;0.089)
		Old	Academic	-	-
			Non-Academic	-0.012	0.031
				(-0.033;0.005)	(-0.005;0.070)
	Male	Young	Academic	0.014	-0.040
				(-0.055;0.093)	(-0.096;0.022)
			Non-Academic	-0.004	-0.004
				(-0.022;0.013)	(-0.037;0.029)
		Middle	Academic	-0.044	-0.052
				(-0.109;0.031)	(-0.120;0.020)
			Non-Academic	-0.008	0.035
				(-0.027;0.009)	(-0.006;0.080)
		Old	Academic	0.004	0.054
				(-0.077;0.110)	(-0.037;0.150)
			Non-Academic	-0.013	0.070
				(-0.032;0.002)	(0.030;0.115)
Moroccan 1st Gen.	Female	Young	Academic	-0.070	0.010
				(-0.158;0.023)	(-0.050;0.075)
			Non-Academic	-0.041	-0.014
				(-0.076;-0.003)	(-0.056;0.028)
		Middle	Academic	0.127	0.094
				(0.044;0.211)	(0.027;0.156)
			Non-Academic	0.013	-0.122
				(-0.029;0.059)	(-0.164;-0.083)
		Old	Academic	-	-
			Non-Academic	0.096	-0.156
				(0.049;0.151)	(-0.195;-0.122)
	Male	Young	Academic	-0.096	0.077
				(-0.188;0.001)	(0.014;0.142)
			Non-Academic	-0.013	0.031
				(-0.047;0.023)	(-0.013;0.077)
		Middle	Academic	0.038	0.088
				(-0.042;0.124)	(0.030;0.147)
			Non-Academic	-0.013	-0.037
				(-0.052;0.031)	(-0.084;0.012)

Table continues on next page ...

Continuation of Table					
**Migration Background**	**Gender**	**Age**	**Education**	**SA**	**UI**
		Old	Academic	0.149 (0.033;0.252)	0.080 (-0.009;0.172)
			Non-Academic	0.005 (-0.033;0.046)	-0.004 (-0.052;0.046)
Moroccan 2nd Gen.	Female	Young	Academic	-0.013 (-0.072;0.053)	0.045 (-0.009;0.102)
			Non-Academic	0.019 (-0.003;0.043)	0.025 (-0.010;0.062)
		Middle	Academic	-	-
			Non-Academic	0.049 (0.020;0.078)	0.021 (-0.020;0.063)
		Old	Academic	-	-
			Non-Academic	-	-
	Male	Young	Academic	0.097 (0.031;0.168)	0.013 (-0.039;0.068)
			Non-Academic	0.075 (0.047;0.107)	-0.001 (-0.035;0.035)
		Middle	Academic	-	-
			Non-Academic	0.074 (0.043;0.107)	0.029 (-0.017;0.074)
		Old	Academic	-	-
			Non-Academic	-	-
Other 1st Gen.	Female	Young	Academic	-0.204 (-0.281;-0.116)	0.009 (-0.047;0.065)
			Non-Academic	0.026 (-0.017;0.071)	-0.075 (-0.107;-0.042)
		Middle	Academic	-0.032 (-0.120;0.057)	0.017 (-0.042;0.075)
			Non-Academic	-0.010 (-0.053;0.034)	-0.087 (-0.124;-0.044)
		Old	Academic	0.032 (-0.055;0.125)	0.009 (-0.048;0.066)
			Non-Academic	0.032 (-0.014;0.080)	-0.114 (-0.145;-0.078)
	Male	Young	Academic	-0.070 (-0.165;0.023)	-0.052 (-0.107;0.004)
			Non-Academic	0.012 (-0.027;0.051)	-0.056 (-0.092;-0.020)
		Middle	Academic	0.163 (0.078;0.251)	0.006 (-0.056;0.071)
			Non-Academic	0.026 (-0.015;0.067)	-0.063 (-0.107;-0.021)
		Old	Academic	0.204 (0.128;0.285)	0.036 (-0.022;0.093)
			Non-Academic	0.019 (-0.023;0.062)	-0.026 (-0.066;0.020)
Other 2nd Gen.	Female	Young	Academic	0.007 (-0.056;0.073)	0.004 (-0.046;0.058)
			Non-Academic	0.009 (-0.010;0.030)	0.036 (0.000;0.074)
		Middle	Academic	-0.080 (-0.130;-0.025)	0.007 (-0.049;0.063)
			Non-Academic	-0.008 (-0.025;0.009)	0.028 (-0.013;0.071)
		Old	Academic	-0.057 (-0.106;-0.006)	0.049 (-0.006;0.106)
			Non-Academic	-0.015 (-0.030;0.001)	0.028 (-0.012;0.071)
	Male	Young	MarrocanAcademic	-0.002	-0.025

Table continues on next page ...

Continuation of Table					
**Migration Background**	**Gender**	**Age**	**Education**	**SA**	**UI**
				(-0.052;0.059)	(-0.073;0.027)
			Non-Academic	-0.005	-0.022
				(-0.019;0.011)	(-0.053;0.015)
		Middle	Academic	-0.037	-0.039
				(-0.085;0.018)	(-0.094;0.017)
			Non-Academic	-0.001	0.028
				(-0.016;0.014)	(-0.018;0.073)
		Old	Academic	0.013	0.079
				(-0.044;0.078)	(0.023;0.136)
			Non-Academic	-0.005	0.096
				(-0.019;0.012)	(0.047;0.150)
Surinamese 1st Gen.	Female	Young	Academic	-0.063	-0.030
				(-0.134;0.014)	(-0.087;0.030)
			Non-Academic	0.005	0.043
				(-0.023;0.034)	(-0.003;0.091)
		Middle	Academic	-0.097	-0.034
				(-0.172;-0.017)	(-0.093;0.024)
			Non-Academic	0.024	0.000
				(-0.014;0.061)	(-0.048;0.052)
		Old	Academic	-0.062	0.006
				(-0.154;0.034)	(-0.061;0.079)
			Non-Academic	-0.003	-0.026
				(-0.039;0.026)	(-0.069;0.016)
	Male	Young	Academic	-0.087	-0.079
				(-0.153;-0.012)	(-0.137;-0.017)
			Non-Academic	0.017	-0.020
				(-0.008;0.043)	(-0.063;0.027)
		Middle	Academic	-0.013	-0.060
				(-0.090;0.074)	(-0.118;0.005)
			Non-Academic	0.018	-0.020
				(-0.012;0.048)	(-0.072;0.030)
		Old	Academic	0.025	-0.028
				(-0.060;0.110)	(-0.092;0.033)
			Non-Academic	0.007	0.010
				(-0.023;0.035)	(-0.035;0.059)
Surinamese 2nd Gen.	Female	Young	Academic	-0.004	0.074
				(-0.068;0.067)	(0.019;0.129)
			Non-Academic	0.034	0.060
				(0.012;0.058)	(0.017;0.104)
		Middle	Academic	-0.061	-0.058
				(-0.122;0.005)	(-0.114;-0.002)
			Non-Academic	0.000	0.007
				(-0.020;0.021)	(-0.038;0.053)
		Old	Academic	-	-
			Non-Academic	-0.004	0.044
				(-0.024;0.017)	(-0.003;0.094)
	Male	Young	Academic	-0.005	-0.028
				(-0.063;0.061)	(-0.080;0.026)
			Non-Academic	0.018	-0.006
				(-0.001;0.038)	(-0.044;0.032)
		Middle	Academic	-0.025	-0.077
				(-0.089;0.046)	(-0.137;-0.018)
			Non-Academic	0.042	0.013
				(0.018;0.069)	(-0.034;0.060)
		Old	Academic	-	-
			Non-Academic	-0.008	0.013
				(-0.026;0.010)	(-0.038;0.064)
Turkish 1st Gen.	Female	Young	Academic	-0.114	0.073
				(-0.185;-0.036)	(0.014;0.135)
			Non-Academic	0.003	0.023
				(-0.026;0.035)	(-0.018;0.067)
		Middle	Academic	-0.089	0.046
				(-0.170;-0.001)	(-0.015;0.106)

Table continues on next page ...

Continuation of Table					
**Migration Background**	**Gender**	**Age**	**Education**	**SA**	**UI**
			Non-Academic	0.037<br>(0.001;0.078)	-0.027<br>(-0.072;0.018)
		Old	Academic	-	-
			Non-Academic	0.063<br>(0.026;0.102)	-0.098<br>(-0.133;-0.062)
	Male	Young	Academic	-0.090<br>(-0.161;-0.016)	0.043<br>(-0.015;0.102)
			Non-Academic	-0.035<br>(-0.058;-0.012)	0.058<br>(0.014;0.102)
		Middle	Academic	0.064<br>(-0.021;0.148)	0.041<br>(-0.018;0.102)
			Non-Academic	0.009<br>(-0.021;0.042)	0.028<br>(-0.021;0.079)
		Old	Academic	0.108<br>(-0.009;0.227)	0.213<br>(0.128;0.298)
			Non-Academic	0.023<br>(-0.005;0.055)	0.024<br>(-0.019;0.071)
Turkish 2nd Gen.	Female	Young	Academic	0.123<br>(0.057;0.195)	0.050<br>(-0.003;0.106)
			Non-Academic	0.043<br>(0.025;0.062)	0.050<br>(0.014;0.092)
		Middle	Academic	-0.005<br>(-0.061;0.076)	0.040<br>(-0.049;0.135)
			Non-Academic	0.073<br>(0.050;0.099)	0.060<br>(0.016;0.108)
		Old	Academic	-	-
			Non-Academic	-	-
	Male	Young	Academic	0.079<br>(0.022;0.147)	-0.053<br>(-0.100;0.001)
			Non-Academic	0.049<br>(0.031;0.070)	0.031<br>(-0.007;0.073)
		Middle	Academic	0.040<br>(-0.027;0.133)	-0.035<br>(-0.120;0.055)
			Non-Academic	0.060<br>(0.041;0.084)	0.004<br>(-0.042;0.050)
		Old	Academic	-	-
			Non-Academic	-	-
