# Supplementary material for: Intersectionality and benefit receipt: The interplay between education, gender, age and migration background
Source: PLoS One. 2024 Nov 14;19(11):e0311241. doi: 10.1371/journal.pone.0311241 (PMC11563431; doi:10.1371/journal.pone.0311241)
Supplement: S3 File — (PDF) [file pone.0311241.s003.pdf]

S3 Additional Analyses

In order to examine the relationship between the length of the observation period and the incidence of benefit receipt, we conducted two distinct analyses. One utilized individual-level data, while the other employed stratum-level data, adhering to the specified analytical sample as outlined in the methods section.

S3.1 Variation in the length of the observation period

We find some variation in the average duration of the observation perioder between intersectional strata, ranging from 5.997 to 11.697 years. Fig S1 illustrates the distribution of average observation periods within strata per age group. The primary source of variation among intersectional groups arises from distinct age brackets within the age groups. Specifically, the middle-age group exhibits a maximum observation period duration of 14 years, whereas younger and older age groups have a maximum observation period of 10 years.

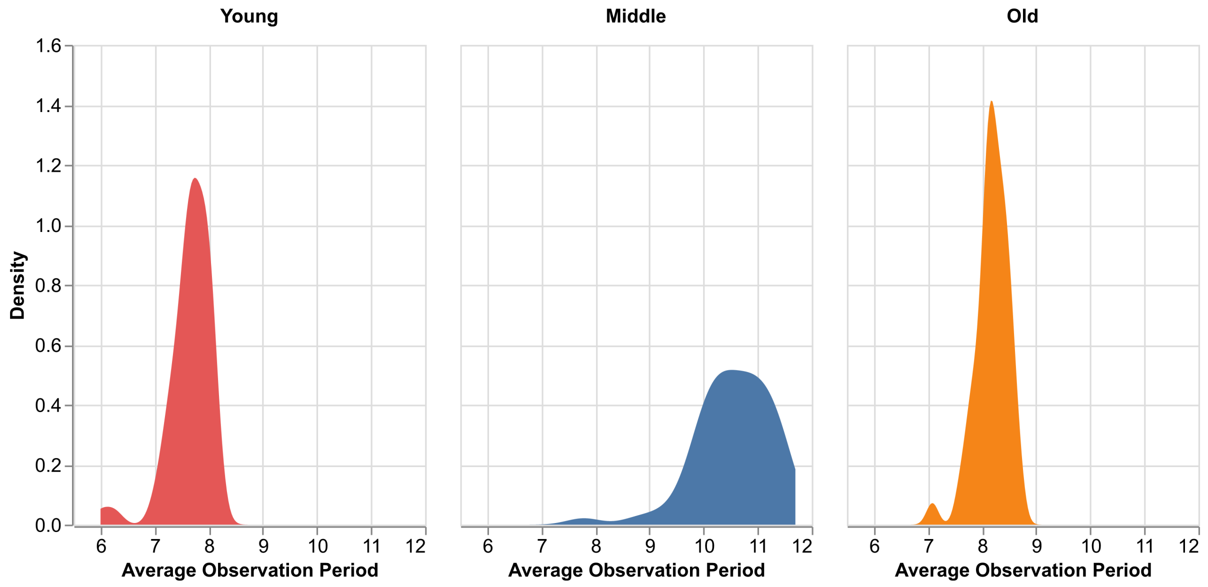

Figure S1: Variation in the average duration of the observation period per stratum by age

Note: Kernel density calculated on the average duration of the the observation period per stratum N(stratum) = 163. **Source:** Authors' own calculation based on non-public individual level register data from the Social Statistical Database (SSD) of Statistics Netherlands (CBS)

Noteworthy instances of brief observation periods include young individuals without an academic degree and a first-generation Eastern European migration background, with the shortest periods recorded (M = 5.997, s.d. = 2.216; M = 6.260, s.d. = 2.238 for men and women, respectively). Similarly, middle-aged first-generation Eastern European men without an academic degree show relatively short

observation periods (M = 7.783, s.d. = 2.938). The brevity of their observations is understandable, given that these groups predominantly consist of labor migrants temporarily residing in the Netherlands. In theory, the shorter observation periods for these groups would lead to a bias favoring lower incidence rates. Nevertheless, we observe middle aged individuals to have higher incidence rates of benefit receipt.

S3.2 The association between incidence and duration of the observation period

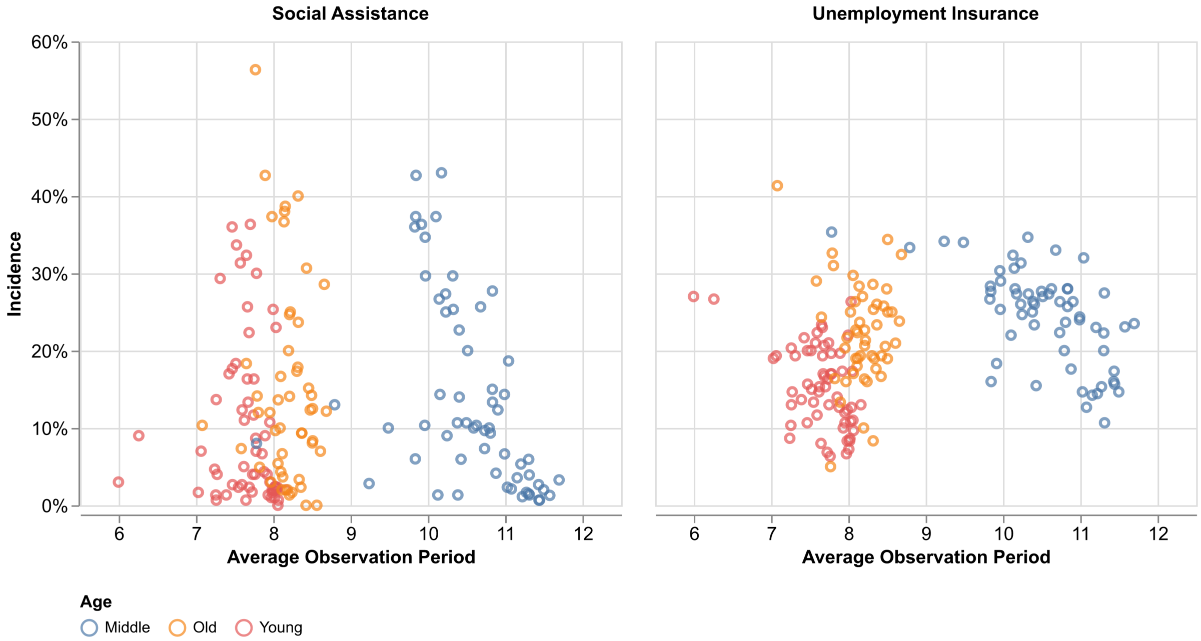

Figure S2: Association Between Benefit Receipt Incidence and Average duration of Observation Period

Source: Authors' own calculation based on non-public individual level register data from the Social Statistical Database (SSD) of Statistics Netherlands (CBS)

Fig S2, illustrates the relationship between the average duration of the observation period and the incidence rates of either social assistance or unemployment insurance. Further analysis, presented in Table S4, corroborates this absence of association for social assistance. However, our analysis does reveal a weak association between the observation period and unemployment insurance incidence. While our initial analysis suggested a relationship between observation period duration and unemployment insurance incidence, this association becomes non-significant when controlling for age differences, see Table S5. This implies that the observed association might be primarily driven by age rather than the duration itself. Nonetheless, the findings presented in the paper might slightly overestimate the unemployment insurance incidence amongst middle-aged individuals.

Table S4: Regression analyses of the association between the duration of the observation period and benefit receipt incidence

	Individual Level		Stratum Level	
	SA	UI	SA	UI
Intercept	0.129	0.127	0.144	0.076
	(0.107; 0.150)	(0.112; 0.141)	(0.023; 0.267)	(0.007; 0.149)
Duration	0.000	0.010	-0.002	0.015
	(-0.001; 0.001)	(0.009; 0.011)	(-0.015; 0.012)	(0.007; 0.022)
Log Likelihood	-12406.061	-22357.746	116.683	211.986
	(-12424.877; -12389.036)	(-22376.611; -22340.648)	(113.515; 118.150)	(208.785; 213.519)
var(sigma strata)	0.015	0.004		
	(0.012; 0.018)	(0.003; 0.006)		
var(sigma)	0.101	0.158	0.015	0.005
	(0.100; 0.103)	(0.156; 0.160)	(0.012; 0.019)	(0.004; 0.006)

Note: Averages of posterior distributions. 95%CI between parentheses. SA = Social Assistance, UI = Unemployment Insurance. N(strata) = 164. Source: Authors' own calculation based on non-public individual level register data from the FD-trygd database provided by Statistics Norway (SSB).

Table S5: Regression analyses of the association between the duration of the observation period and benefit receipt incidence controlled for age

	Individual Level		Stratum Level	
	SA	UI	SA	UI
Intercept	0.141	0.157	0.740	0.789
	(0.110; 0.172)	(0.155; 0.159)	(0.379; 1.104)	(0.611; 0.965)
Duration	0.000	0.001	-0.057	-0.052
	(-0.001; 0.001)	(-0.000; 0.002)	(-0.091; -0.023)	(-0.068; -0.035)
Young	0.015	-0.000	-0.120	-0.144
	(-0.031; 0.054)	(-0.025; 0.025)	(-0.214; -0.024)	(-0.189; -0.098)
Old	-0.038	-0.065	-0.202	-0.240
	(-0.079; 0.006)	(-0.088; -0.041)	(-0.311; -0.091)	(-0.292; -0.186)
Log Likelihood	-12406.390	-22358.330	124.139	252.315
	(-12441.790; -12376.810)	(-22393.720; 22326.550)	(120.065; 126.399)	(248.235; 254.608)
var(sigma strata)	0.014	0.003		
	(0.011; 0.018)	(0.003; 0.005)		
var(sigma)	0.101	0.158	0.014	0.003
	(0.100; 0.103)	(0.156; 0.160)	(0.011; 0.017)	(0.003; 0.004)

Note: Averages of posterior distributions. 95%CI between parentheses. N(strata) = 164. Source: Authors' own calculation based on non-public individual level register data from the FD-trygd database provided by Statistics Norway (SSB).
